# Endocannabinoid-LTP Mediated by CB1 and TRPV1 Receptors Encodes for Limited Occurrences of Coincident Activity in Neocortex

**DOI:** 10.3389/fncel.2018.00182

**Published:** 2018-07-05

**Authors:** Yihui Cui, Sylvie Perez, Laurent Venance

**Affiliations:** Center for Interdisciplinary Research in Biology (CIRB), College de France, INSERM U1050, CNRS UMR7241, Paris Sciences et Lettres Research University, Paris, France

**Keywords:** endocannabinoids, spike-timing dependent plasticity, neocortex, LTP, CB_1_R, TRPV1, Hebbian plasticity

## Abstract

Synaptic efficacy changes, long-term potentiation (LTP) and depression (LTD), underlie various forms of learning and memory. Synaptic plasticity is generally assessed under prolonged activation, whereas learning can emerge from few or even a single trial. Here, we investigated the existence of rapid responsiveness of synaptic plasticity in response to a few number of spikes, in neocortex in a synaptic Hebbian learning rule, the spike-timing-dependent plasticity (STDP). We investigated the effect of lowering the number of pairings from 100 to 50, and 10 on STDP expression, using whole-cell recordings from pyramidal cells in rodent somatosensory cortical brain slices. We found that a low number of paired stimulations induces LTP at neocortical layer 4–2/3 synapses. Besides the asymmetric Hebbian STDP reported in the neocortex induced by 100 pairings, we observed a symmetric anti-Hebbian LTD for 50 pairings and unveiled a unidirectional Hebbian spike-timing-dependent LTP (tLTP) induced by 10–15 pairings. This tLTP was not mediated by NMDA receptor activation but requires CB_1_ receptors and transient receptor potential vanilloid type-1 (TRPV1) activated by endocannabinoids (eCBs). eCBs have been widely described as mediating short- and long-term synaptic depression. Here, the eCB-tLTP reported at neocortical synapses could constitute a substrate operating in the online learning of new associative memories or during the initial stages of learning. In addition, these findings should provide useful insight into the mechanisms underlying eCB-plasticity occurring during marijuana intoxication.

## Introduction

In mammals, cardinal cognitive abilities can display very rapid learning dynamics. Forming new associative memories and behavioral rules can be learned within a few or even a single trial (Schultz et al., 2003; Pasupathy and Miller, 2005; Armstrong et al., 2006; Rutishauser et al., 2006; Whitlock et al., 2006; Tse et al., 2007; Quilodran et al., 2008; Cook and Fagot, 2009; Ito and Doya, 2009; Izquierdo et al., 2016). Cortical and striatal neurons that respond to relevant cues, actions or rewards fire few action potentials (~1–12) upon each trial (Schultz et al., 2003; Pasupathy and Miller, 2005; Quilodran et al., 2008). This indicates that the emission of a low number of action potentials should be sufficient to allow synaptic plasticity expression. However, common cellular conditioning protocols, such as high- or low-frequency stimulations, used for the induction of long-term plasticity involve hundreds (or even more than one thousand) of pre- and/or postsynaptic action potentials. Noticeable exceptions are studies reporting that single-shock synaptic stimulation of layer 5 neocortical pyramidal neurons induced NMDAR-dependent long-term depression (LTD) in visual cortex (Holthoff et al., 2004) and that single-burst of strong and synchronous inputs from hippocampal CA1 to CA3 triggered NMDAR- and L-type voltage-sensitive calcium channels (VSCCs) dependent long-term potentiation (LTP; Remy and Spruston, 2007). Moreover, it was shown that low numbers of paired stimulations (~20) in spike-timing-dependent plasticity (STDP) paradigm (Dan and Poo, 2006; Sjöström et al., 2008; Feldman, 2012), were able to induce spike-timing-dependent potentiation (tLTP) in dissociated culture of hippocampal neurons (Zhang et al., 2009), in cortical slices (Froemke et al., 2006) and in corticostriatal slices (Cui et al., 2015, 2016). These studies revealed that limited occurrences of coincident activity are able to induce bidirectional plasticity, and this needs to be extended to other synapses and cell conditioning paradigms.

We previously reported in striatum the existence of an endocannabinoid-mediated spike-timing dependent LTP (eCB-tLTP) induced with a very low number of pairings (from 5 to 15 pairings; Cui et al., 2015, 2016). STDP is a synaptic Hebbian learning rule in which synaptic weight changes depend on the activity on both sides of the synapse (Dan and Poo, 2006; Sjöström et al., 2008; Feldman, 2012). Since its discovery, STDP has attracted considerable interest in experimental and in computational neuroscience because it relies on spike correlation and has emerged as a candidate mechanism for activity-dependent changes in neural circuits, including map plasticity (Abbott and Nelson, 2000; Dan and Poo, 2006; Morrison et al., 2008; Sjöström et al., 2008; Feldman, 2012; Froemke, 2015; Korte and Schmitz, 2016). Here, we tested at neocortical layer 2/3 synapses the hypothesis that a low number of spikes (~10–15) could lead to long-term synaptic plasticity accounting for fast and flexible learning of new behavioral responses. At neocortical layer 4-2/3 synapses, a hundred of pairings induced Hebbian STDP, i.e., tLTP and tLTD being triggered by causal pre-before-postsynaptic pairings and anti-causal post-before-presynaptic pairings, respectively (Feldman, 2000; Bender K. J. et al., 2006; Bender V. A. et al., 2006; Froemke et al., 2006; Nevian and Sakmann, 2006; Banerjee et al., 2009; Itami and Kimura, 2012; Rodríguez-Moreno et al., 2013; Banerjee et al., 2014). STDP polarity was developmentally controlled (Banerjee et al., 2009; Itami and Kimura, 2012, 2016). In the present study, we observed that STDP polarity changed depending on the number of pairings: from asymmetric Hebbian STDP for 100 pairings, to symmetric anti-Hebbian tLTD for 50 pairings and to unidirectional tLTP for 10–15 pairings. Notably, a low number of paired stimulations (~10–15) were sufficient to trigger tLTP. We found that this tLTP displays an unidirectional Hebbian polarity. This tLTP was not NMDAR-dependent but eCB-mediated and required the activation of CB_1_ receptors and transient receptor potential vanilloid type-1 (TRPV1). Our study evidences, together with recent reports (Cui et al., 2015, 2016; Wang et al., 2016, 2018; Maglio et al., 2018) that eCB system not only promotes LTD but also LTP. Therefore, endocannabinoids (eCBs) can underlie bidirectional plasticity, depending on the regime of activity pattern on both sides of the synapse.

## Materials and Methods

### Animal Models

All experiments were performed in accordance with the guidelines of the local animal welfare committee (Center for Interdisciplinary Research in Biology Ethics Committee) and the EU (directive 2010/63/EU). Every precaution was taken to minimize stress and the number of animals used in each series of experiments. Animals were housed in standard 12 h light/dark cycles and food and water were available *ad libitum*. Sprague-Dawley rats (Charles River, L’Arbresle, France) and C57BL/6 mice type-1 cannabinoid receptor knockout, (CB_1_R^−/–^), and wild-type littermates (CB_1_R^+/+^) mice (Ledent et al., 1999), were used for *ex vivo* electrophysiology.

### Whole-Cell Patch-Clamp Recordings

Horizontal brain slices containing the somatosensory cortex with a thickness of 330 or 300 μm were prepared, respectively, from postnatal day 25–35 rats or mice using a vibrating blade microtome (VT1200S, Leica Micosystems, Nussloch, Germany). Brains were sliced in a 95% CO_2_/5% O_2_-bubbled, ice-cold cutting solution containing (in mM): 125 NaCl, 2.5 KCl, 25 glucose, 25 NaHCO_3_, 1.25 NaH_2_PO_4_, 2 CaCl_2_, 1 MgCl_2_, 1 pyruvic acid, and then transferred into the same solution at 34°C for 1 h and then moved to room temperature. Patch-clamp recordings were performed as previously described (Cui et al., 2015). Whole-cell recordings borosilicate glass pipettes of 4–6 MΩ resistance were filled with (in mM): 122 K-gluconate, 13 KCl, 10 phosphocreatine, 10 HEPES, 4 ATP-Mg, 0.3 GTP-Na, 0.3 EGTA (adjusted to pH 7.35 with KOH). The composition of the extracellular solution was (in mM): 125 NaCl, 2.5 KCl, 25 NaHCO_3_, 2 CaCl_2_, 1 MgCl_2_, 1.25 NaH_2_PO_4_, 25 glucose, 10 μM pyruvic acid bubbled with 95% O_2_ and 5% CO_2_. Signals were amplified using EPC10-2 amplifiers (HEKA Elektronik, Lambrecht, Germany). All recordings were performed at 34°C using a temperature control system (Bath-controller V, Luigs&Neumann, Ratingen, Germany) and slices were continuously superfused at 2–3 ml/min with the extracellular solution. Series resistance was not compensated. Recordings were filtered at 5 kHz and sampled at 10 kHz with the Patchmaster v2 x 32 program (HEKA Elektronik).

### Chemicals

DL-2-amino-5-phosphono-pentanoic acid (D-AP5, 50 μM; Tocris, Ellisville, MO, USA) and 2-Methyl-6-(phenylethynyl) pyridine hydrochloride (MPEP hydrochloride, 10 μM; Tocris) were dissolved directly in the extracellular solution and bath applied. N-(piperidin-1-yl)-5-(4-iodophenyl)-1-(2,4-dichlorophenyl)-4-methyl-1H-pyrazole-3-carboxamide (AM251, 3 μM; Tocris), picrotoxin (50 μM; Sigma), 1,4-Dihydro-2,6-dimethyl-4-(3-nitrophenyl)-3,5-pyridinedicarboxylic acid 2-methyloxyethyl 1-methylethyl ester (nimodipine, 1 μM; Tocris) and (2*E*)-*N*-(2,3-Dihydro-1,4-benzodioxin-6-yl)-3-[4-(1,1-dimethylethyl)phenyl]-2-propenamide (AMG9810, 1 μM; Tocris) were dissolved in ethanol and then added in the external solution at a final concentration of ethanol of 0.01%–0.1%. N-[2-(4-Chlorophenyl)ethyl]-1,3,4,5-tetrahydro-7,8-dihydroxy-2H-2-benzazepine-2-carbothioamide (capsazepine, 10 μM; Tocris) and (E)-N-[(4-hydroxy-3-methoxyphenyl)methyl]-8-methyl-6-nonenamide (capsaicin, 10 μM; Tocris) were dissolved in DMSO and then added in the external solution at a final concentration of DMSO of 0.001 and 0.0025, respectively. Tetrahydrolipstatin (THL, 10 μM; Sigma) was dissolved in DMSO (0.08%) and applied internally via the patch-clamp pipette. DMSO (0.08%), the vehicle used to dilute i-THL, did not preclude the tLTP induced with 10 post-pre pairings (Cui et al., 2015).

### Spike-Timing-Dependent Plasticity Induction Protocols

**Figure 1:**
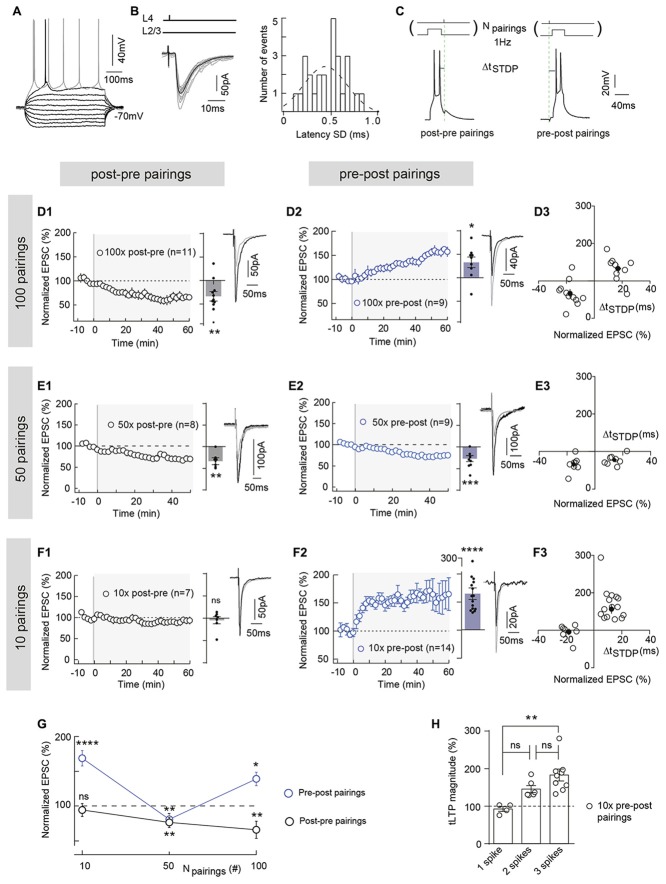
Polarity of spike-timing dependent plasticity (STDP) varies upon number of pairings (*N*_pairings_) and *N*_pairings_ = 10 induce spike-timing-dependent long-term potentiation (tLTP) in neocortical layer 2/3. (**A**) Characteristic voltage responses of a pyramidal cell to a series of 500 ms current pulses from −150 to +90 pA with current steps increasing by 30 pA (black traces) and to +60 pA above spike threshold (gray trace). **(B)** Cortically-evoked pyramidal cell EPSCs (averages of 15 traces; left panel). Distribution of latency SD, centered on 0.47 ms and fitted by a Gaussian function, indicate a monosynaptic cortical transmission because inferior to 1 ms (right panel). **(C)** Experimental design of STDP paired stimulations: two spikes evoked in pyramidal cell were paired with a single cortical layer 4 electrical stimulation; this pairing being repeated 10, 50 or 100 times at 1 Hz. Δt_STDP_ indicates the time between pre- and postsynaptic stimulations. Δt_STDP_ < 0 and Δt_STDP_ > 0 refer to post-pre and pre-post pairings, respectively. **(D)** 100 pairings induced bidirectional Hebbian plasticity at neocortical layer 2/3 synapses. **(D1,D2)** Summary of timing-dependent long-term depression (tLTD) and tLTP induced by 100 post-pre (*n* = 11 cells from 8 rats; Δt_STDP_ = −17 ± 2 ms) **(D1)** and 100 pre-post pairings (*n* = 9 cells from 6 rats; Δt_STDP_ = +17 ± 2 ms) **(D2)**, respectively. **(D3)** Graph summarizing STDP (bidirectional Hebbian) occurrence for 100 pairings. **(E)** 50 pairings induced symmetric anti-Hebbian tLTD. **(E1,E2)** Summary of STDP experiments showing tLTD for both post-pre (*n* = 8 cells from 5 rats; Δt_STDP_ = −15 ± 1 ms) **(E1)** and pre-post (*n* = 9 cells from 5 rats; Δt_STDP_ = +14 ± 2 ms) **(E2)** pairings. **(E3)** Graph summarizing STDP (symmetric anti-Hebbian tLTD) occurrence for 50 pairings. **(F)** 10 pairings induced unidirectional Hebbian plasticity. **(F1,F2)** Summary of STDP experiments showing the absence of STDP and tLTP induced by 10 post-pre (*n* = 7 cells from 3 rats; Δt_STDP_ = −16 ± 2 ms) **(F1)** and 10 pre-post (*n* = 15 cells from 6 rats; Δt_STDP_ = +13 ± 1 ms) **(F2)** pairings, respectively. **(F3)** Graph summarizing STDP (unidirectional Hebbian) occurrence for 10 pairings. **(G)** Summary graph illustrating the cortical STDP expression and polarity for 100, 50 and 10 post-pre and pre-post pairings. **(H)** Relationship between the number of postsynaptic action potentials (per pairing) and the synaptic efficacy changes after 10 pre-post pairings (one way analyses of variance (ANOVA) test, *p* = 0.0027, *F* = 8.794. 1 spike vs. 2 spikes, *p* = 0.0892; 2 spikes vs. 3 spikes, *p* = 0.1545; 1 spike vs. 3 spikes, *p* = 0.0020). Representative traces are the average of 15 EPSCs during baseline (black traces) and 50 min after STDP protocol (gray traces). Error bars represent SD. *p < 0.05; **p < 0.01; ^***^p < 0.005; ***p < 0.0001. ns, not significant.

Electrical stimulation was performed with a bipolar electrode (Phymep, Paris, France) placed in the layer 4 of the somatosensory cortex. Electrical stimulation was monophasic at constant current (ISO-Flex stimulator, AMPI, Jerusalem, Israel). Currents were adjusted to evoke 50–250 pA excitatory postsynaptic currents (EPSCs). Repetitive control stimuli were applied at 0.1 Hz. STDP protocols consisted of pairings of pre- and postsynaptic stimulations (100, 50 or 10 at 1 Hz) separated by a specific time interval (Δt_STDP_); Δt_STDP_ was estimated as the time interval between the stimulation artifact recorded in the postsynaptic cell and the neighboring postsynaptic action potential. Presynaptic stimulation corresponded to cortical layer 4 stimulation and the postsynaptic stimulation to an action potential evoked by a depolarizing current step for 30 ms duration (injected currents were 370 ± 35 pA for the 10 pre-post pairing experiments, *n* = 14) in one layer 2/3 pyramidal cell. Δt_STDP_ < 0 ms for post-pre (post-before-pre) pairings, and Δt_STDP_ > 0 ms for pre-post (pre-before-post) pairings. Pyramidal cells were maintained during the whole duration of the experiments at a constant holding membrane potential which corresponds to their initial resting membrane potential (−66 ± 1 mV, *n* = 40). Thus, EPSCs during baseline or after STDP protocol were measured at the same membrane potential (in voltage-clamp mode); STDP pairings (performed in current-clamp mode) were conducted also at this same holding membrane potential. A single STDP protocol was applied per cell, and only one cell was recorded per brain slice. Neuronal recordings were made over a period of 10 min at baseline, and for 60 min after the STDP protocols; long-term changes in synaptic efficacy were measured from 45 min to 55 min. We individually measured and averaged 60 successive EPSCs, comparing the last 10 min of the recording with the 10-min baseline recording. Experiments were excluded if input resistance (Ri) varied by more than 20%. After recording of 10 min control baseline, drugs were applied in the bath. A new baseline with drugs was recorded after a time lapse of 10 min (to allow the drug to be fully perfused) for 10 min before the STDP protocol. Drugs were present until the end of the recording; except for picrotoxin, which was bath-applied 40 min after pairing protocol. In a subset of experiments, THL were applied intracellularly via the patch-clamp pipette (i-THL). Once the cell patched, drugs were allowed to diffuse into the cell during at least 10 min before starting recording of the baseline. STDP protocols consisting of 10 pre-post pairings (with 2–3 postsynaptic spikes) were sufficient to induce potent tLTP in rats whereas in C57BL/6 mice 15 pairings (still with 2–3 postsynaptic spikes per postsynaptic discharge) were required to trigger tLTP. Note that 2–3 action potentials per pairing were required for 10 pairings to induce tLTP since single backpropagating action potentials paired with presynaptic stimulation, did not induce plasticity (105 ± 5%, *p* = 0.4029, *n* = 4; Figure 1H).

Post-pre and pre-post Δt_STDP_ were comprised between 5 ms and 20 ms (absolute values) which is within the temporal domain of expression of STDP (Feldman, 2012). We ensured that Δt_STDP_ (absolute) values did not display significant variations among experimental groups for post-pre or pre-post pairing protocol (one-way ANOVA: *F* = 1.782; *p* = 0.0968, Dunnett’s multiple comparisons test; with absolute values of Δt_STDP_; Supplementary Table S1).

### Patch-Clamp Data Analysis

Off-line analysis was performed using Fitmaster (Heka Elektronik) and Igor-Pro 6.0.3 (Wavemetrics, Lake Oswego, OR, USA). Statistical analysis was performed using Prism 5.0 (San Diego, CA, USA). “n” refers to an experiment on a single cell from a single brain slice (the number of animals for each experimental group are indicated in the legends of the figures). Experimenters were blind to the genotype of *CB*_1_*R*^−/–^ and *CB*_1_*R*^+/+^ littermate mice. All results are expressed as mean ± SEM in the text and as mean ± SD for visualization purposes in the figures, and statistical significance was assessed using two-sided Student’s *t* test or the one sample *t* test as appropriate using the indicated significance threshold (*p*). We analyzed all datasets (Prism 6.0 software) and all of them fitted Gaussian distribution with equal variance.

Plasticity loci were determined using the mean variance analysis method (Clements and Silver, 2000). EPSC coefficient of variation (CV) was calculated by the ratio of the SD and the mean EPSC amplitude during the 10 min of baseline and after STDP protocol (for 10 min, between 40 min and 50 min after pairings). The plasticity locus was then deduced from the relationship between the normalized CV^−2^ (CV^−2^ between 40 min and 50 min after pairing protocol /CV^−2^ during baseline) and the normalized EPSC amplitudes (EPSC mean amplitude after induction of plasticity, between 40 min and 50 min after pairing protocol /EPSC mean amplitude during baseline). Normalized CV^−2^ ≥ normalized EPSCs amplitude indicates mainly presynaptic modifications, whereas normalized CV^−2^ < normalized EPSC amplitude reflects a mixed (post- and presynaptic) loci of plasticity. The case where an absence of variation of normalized CV^−2^ is associated with a variation of normalized EPSC amplitude reflects mainly postsynaptic modifications (Clements and Silver, 2000).

## Results

### Polarity of STDP Varies Upon Number of Pairings at Neocortical Layer 4-2/3 Synapses

To examine the effect of a few number of pairings on long-term synaptic efficacy changes, we made whole-cell recordings from pyramidal cells of the somatosensory cortex in horizontal brain slices (Figure 1A). In cortical slices, layer 2/3 pyramidal cells receive monosynaptic inputs from the layer 4 as illustrated by EPSC latency standard deviation (0.47 ± 0.04 ms, *n* = 26), which is inferior to 1 ms (Figure 1B). We investigated the effect of lowering the number of pairings from 100 to 50 and 10 on STDP (Figure 1). Baseline EPSCs were recorded for 10 min followed by pairing a single presynaptic stimulation with a postsynaptic brief depolarization of the recorded pyramidal cell which induces 2–3 spikes. The STDP protocol consisted in pairing pre- and postsynaptic stimulations with a fixed timing interval, Δt_STDP_ (Δt_STDP_ < 0 indicates that postsynaptic stimulation preceded presynaptic stimulation and Δt_STDP_ > 0 indicates that presynaptic stimulation preceded postsynaptic stimulation), repeated n times at 1 Hz (Figure 1C). Post-pre and pre-post Δt_STDP_ stood between 5 ms and 20 ms (absolute values) i.e., within the temporal domain of expression of neocortical STDP (see in “Materials and Methods” section and Figure Legends for detailed values of Δt_STDP_ for each experimental condition; Dan and Poo, 2006; Sjöström et al., 2008; Feldman, 2012). After the STDP pairings, EPSCs were monitored for 1 h.

In the somatosensory cortex, pyramidal cells exhibit a Hebbian STDP (Markram et al., 1997; Feldman, 2000; Sjöström et al., 2001; Bender K. J. et al., 2006; Bender V. A. et al., 2006; Froemke et al., 2006; Nevian and Sakmann, 2006; Banerjee et al., 2014). Although this Hebbian STDP has been observed using different number and frequency of pairings, it appears that tLTP is NMDAR-mediated whereas t-LTD has been shown to depend either on metabotropic glutamate receptor (mGluR)- and/or CB_1_R or presynaptic NMDAR (Sjöström et al., 2003; Bender V. A. et al., 2006; Nevian and Sakmann, 2006; Corlew et al., 2007; Seol et al., 2007; Rodríguez-Moreno and Paulsen, 2008; Rodríguez-Moreno et al., 2010, 2011; Banerjee et al., 2014; Itami and Kimura, 2016; reviewed in Sjöström et al., 2008; Feldman, 2012). When we paired post- and presynaptic activities 100 times at 1 Hz within a narrow time window (−25 < Δt_STDP_ < +25 ms) in layer 2/3 pyramidal cells, we observed an Hebbian plasticity. An example of tLTD induced by 100 post-pre pairings is illustrated in the Supplementary Figure S1A1; input resistance (Ri) and injected current (Ij) remained stable over this period. Conversely, pre-post pairings induced tLTP as shown in the example in the Supplementary Figure S1A2. To summarize, post-pre pairings induced tLTD (mean EPSC amplitude recorded 60 min after protocol induction: 66.5 ± 10.0% of the baseline, *p* = 0.0073, *n* = 11; 9/11 cells displayed tLTD), whereas pre-post pairings induced tLTP (133.1 ± 11.2%, *p* = 0.0193, *n* = 9; 7/9 cells displayed tLTP; Figures 1D1,D2), resulting in Hebbian STDP (Figures 1D3,G).

Pyramidal cells display multiple forms of STDP, which are induced depending on Δt_STDP_ as aforementioned but also on the number of pairings (*N*_pairings_; Sjöström et al., 2001; Froemke et al., 2006). In the dorsolateral striatum, we recently reported that a very low number of pairings (*N*_pairings_ = 5–15) induced eCB-tLTP, dependent on CB_1_R and TRPV1 activation (Cui et al., 2015, 2016). We investigated here if similar plasticity also exists in neocortex.

We first decreased the number of pairings from 100 to 50 and observed tLTD for both post-pre and pre-post pairings: as exemplified in the Supplementary Figures S1B1,B2, post-pre pairings induced tLTD and pre-post pairings induced tLTD. To summarize, tLTD persisted with post-pre pairings (66.0 ± 7.1%, *p* = 0.0067, *n* = 8; 7/8 cells displayed tLTD; Figure 1E1), whereas the tLTP classically triggered on the pre-post pairing side (Figure 1D2) was flipped into tLTD for 50 pairings (75.4 ± 4.6%, *p* = 0.0007, *n* = 9; 7/9 cells displayed tLTD; Figure 1E2). Thus, bidirectional Hebbian STDP observed with 100 pairings was switched into symmetric anti-Hebbian tLTD for 50 pairings (Figure 1G).

We next lowered the number of pairings down to 10 and observed an unidirectional Hebbian tLTP (Figure 1F). Indeed, as exemplified in the Supplementary Figures S1C1,C2, 10 post-pre pairings did not induce synaptic efficacy changes, whereas 10 pre-post pairings induced tLTP. To summarize, 10 post-pre pairings failed to induce significant long-term plasticity (95.5 ± 7% of the baseline, *p* = 0.5884, *n* = 7; 6/7 cells displayed an absence of significant plasticity; Figure 1F1), whereas 10 pre-post pairings were able to induce a potent tLTP (168.2 ± 11.1%, *p* < 0.0001, *n* = 15; 15/15 cells displayed tLTP; Figures 1F2,3). We analyzed the relationship between the synaptic efficacy changes after 10 pre-post pairings and the EPSC amplitude during the baseline. There was no significant correlation between plasticity and EPSC amplitude (50–260 pA) during baseline (10 pre-post pairings, *R*^2^ = 0.1012, *p* = 0.2478, *n* = 15; Supplementary Figure S1D). Interestingly, 2–3 postsynaptic action potentials (per pairing) were necessary to induce tLTP with 10 pre-post pairings, whereas a single action potential was sufficient to induce tLTD and tLTP with post-pre and pre-post pairings, respectively (Figure 1H). Indeed, when a postsynaptic single action potential was evoked (per pairing), no plasticity was observed (Figure 1H). Accordingly, we have tested the occurrence of plasticity for 10 post-pre pairings in similar conditions than the ones used for pre-post pairings, i.e., with 2–3 postsynaptic action potentials for every pairings.

In conclusion, asymmetric Hebbian STDP (100 pairings) is flipped to symmetric anti-Hebbian tLTD (50 pairings) and unidirectional Hebbian tLTP (10 pairings) with decreasing numbers of paired stimulations (from 100 to 10) at neocortical layer 4-2/3 synapses (Figure 1G).

### 10 Pairings-tLTP Is NMDAR-Independent but Relies on mGluR5 and L-Type VSCC Activation

**Figure 2:**
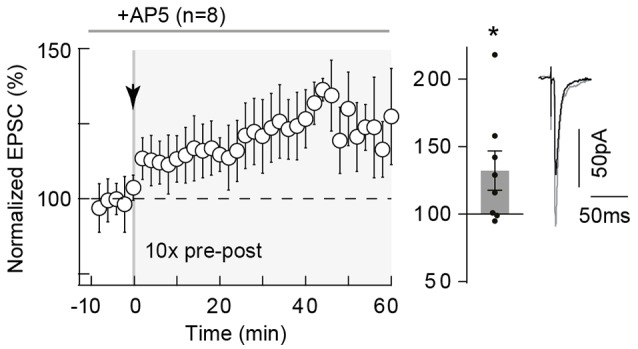
10 pairings-tLTP is NMDAR-independent. 10 pairings-tLTP is NMDAR-independent. Summary of tLTP induced by 10 pre-post pairings with DL-2-amino-5-phosphono-pentanoic acid (D-AP5; 50 μM; *n* = 8 cells from 4 rats; Δt_STDP_ = +10 ± 1 ms). Inhibition of NMDAR with D-AP5 did not prevent the induction of tLTP. Representative traces are the average of 15 excitatory postsynaptic currents (EPSCs) during baseline (black traces) and 50 min after STDP protocol (gray traces). Error bars represent SD. *p < 0.05.

We next questioned the mechanism of tLTP induced by *N*_pairings_ = 10 and first tested whether this tLTP would be NMDAR-mediated. Indeed, neocortical tLTP induced by pre-post pairings (using various frequencies and numbers of pairings) has been reported to be NMDAR-mediated (Sjöström et al., 2003; Bender V. A. et al., 2006; Nevian and Sakmann, 2006; Corlew et al., 2007; Rodríguez-Moreno and Paulsen, 2008; Banerjee et al., 2014; reviewed in Sjöström et al., 2008; Feldman, 2012). For this purpose, we bath-applied the selective NMDAR blocker D-AP5 (50 μM), which had no effect on synaptic transmission (normalized EPSC_(baseline-drugs/baseline-control)_ amplitude: 96.1 ± 1.7%, *p* = 0.0703, *n* = 6). Here, tLTP induced by *N*_pairings_ = 10 was not NMDAR-activation dependent. Indeed, an example of tLTP induced by 10 pre-post pairings in presence of D-AP5 is shown in the Supplementary Figure S2A. To summarize, tLTP induced with 10 pre-post pairings was not significantly affected by D-AP5 (132.3 ± 14.5% of the baseline, *p* = 0.0437, *n* = 8; 5/8 displayed tLTP; Figure 2), questioning the identity of the signaling pathways underlying this tLTP.

**Figure 3:**
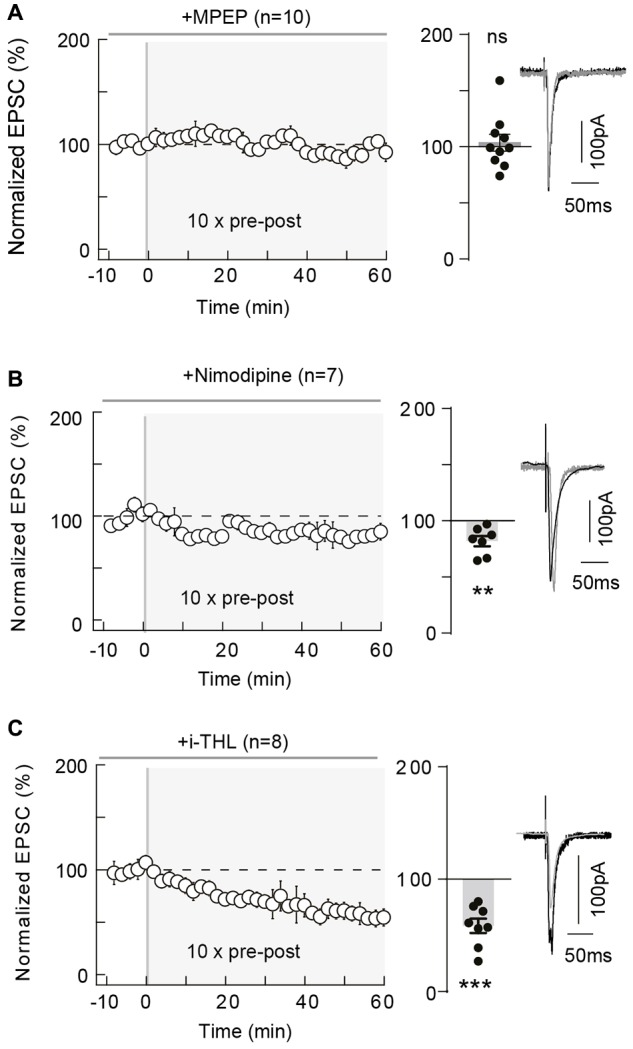
10 pairings-tLTP relies on type-5 metabotropic glutamate receptor (mGluR5) and L-type voltage-sensitive calcium channel (VSCC) activation and involves postsynaptic 2-AG. **(A)** 10 pairings-tLTP is dependent on mGluR5 activation. Summary of experiments showing that tLTP expression was prevented by 2-Methyl-6-(phenylethynyl) pyridine (MPEP; *n* = 10 cells from 4 rats; Δt_STDP_ = +13 ± 1 ms). **(B)** 10 pairings-tLTP is dependent on L-type VSCC activation. Summary of experiments showing that tLTP expression was prevented by nimodipine (*n* = 7 cells from 3 rats; Δt_STDP_ = +11 ± 1 ms). **(C)** 10 pairings-tLTP is dependent on diacylglycerol lipase-α activity. Summary of experiments showing that tLTP expression was prevented by i-THL (*n* = 8 cells from 5 rats; Δt_STDP_ = +14 ± 2 ms). Representative traces are the average of 15 EPSCs during baseline (black traces) and 50 min after STDP protocol (gray traces). Error bars represent SD. **p < 0.01; ^***^p < 0.005. ns, not significant.

Since tLTP induced by 10 pairings in striatum was type-5 metabotropic glutamate receptor (mGluR5) and VSCC dependent (Cui et al., 2015), we tested if these elements were involved in cortical tLTP. In the somatosensory cortex, mGluR5 are expressed in pyramidal cells, where mGluR5 constitute the dominant group I mGluR subtype with a postsynaptic expression (López-Bendito et al., 2002; Wijetunge et al., 2008). In addition, in layer 2/3 pyramidal cells, backpropagating action potentials activate VSCC, which allows large calcium influxes in distal dendritic spines (Koester and Sakmann, 2000). We first tested whether mGluR5 was involved in the 10-pairings-tLTP, by bath-applied MPEP (10 μM), a specific mGluR5 antagonist (Figure 3A). As exemplified in the Supplementary Figure S2B, tLTP was prevented by MPEP treatment. In summary, MPEP prevented the induction of 10 pairings tLTP (103.7 ± 7.5% of the baseline, *p* = 0.6318, *n* = 10; 1/10 cells displayed tLTP; Figure 3A). We next tested a blocker of L-type VSCCs, nimodipine (1 μM), which had no effect on synaptic transmission (normalized EPSC_(baseline-drugs/baseline-control)_ amplitude: 105.1 ± 4.8%, *p* = 0.3289, *n* = 7). We demonstrated that calcium entry via L-type VSCCs was involved in tLTP induced with 10 pre-post pairings because nimodipine precluded tLTP. Indeed, in the Supplementary Figure S2C is shown an example of a lack of plasticity observed with nimodipine application and in summary 10 pre-post pairings failed inducing tLTP with L-type VSCC blockade (81.9 ± 4.6% of the baseline, *p* = 0.0080, *n* = 7; 0/7 cells displayed tLTP; Figure 3B). Thus, 10 pairings-tLTP is NMDAR-independent but mediated by the activation of mGluR5 and L-type VSCCs.

### 10 Pairings-tLTP Involves Postsynaptic 2-AG Signaling and Is CB_1_R-Mediated With a Presynaptic Induction Locus

According to our previous results obtained in striatum (Cui et al., 2015, 2016) and because mGluR5 and VSCCs are involved in eCB synthesis (Piomelli et al., 2007; Di Marzo, 2008; Kano et al., 2009; Alger and Kim, 2011), we then asked whether tLTP induced by 10 pre-post pairings was endocannabinoid- and CB_1_R-mediated.

Concomitant activations of mGluR5 (belonging to Gq/11-coupled receptors, whose stimulation results in PLCβ activation) and VSCC promote diacylglycerol lipase-α activity and therefore 2-arachidonoylglycerol (2-AG) synthesis (Piomelli et al., 2007; Di Marzo, 2008; Kano et al., 2009; Alger and Kim, 2011). 2-AG is produced from the PLCβ product diacylglycerol by calcium-activated diacylglycerol lipase-α and is the principal eCB involved in modulating synaptic weight via CB_1_R activation (Piomelli et al., 2007). We applied intracellularly via the patch-clamp pipette a diacylglycerol lipase-α inhibitor, tetrahydrolipstatin (10 μM, i-THL) and we observed that i-THL prevented tLTP as illustrated in the example in Supplementary Figure S2D and in the summary graph (58.5 ± 6.5%, *p* = 0.0004, *n* = 8; 0/8 cells displayed tLTP; Figure 3C). Because the i-THL application was confined to the recorded neuron, this indicates that the production of 2-AG needed to activate CB_1_R arises from the postsynaptic pyramidal cell subjected to the paired stimulations.

**Figure 4:**
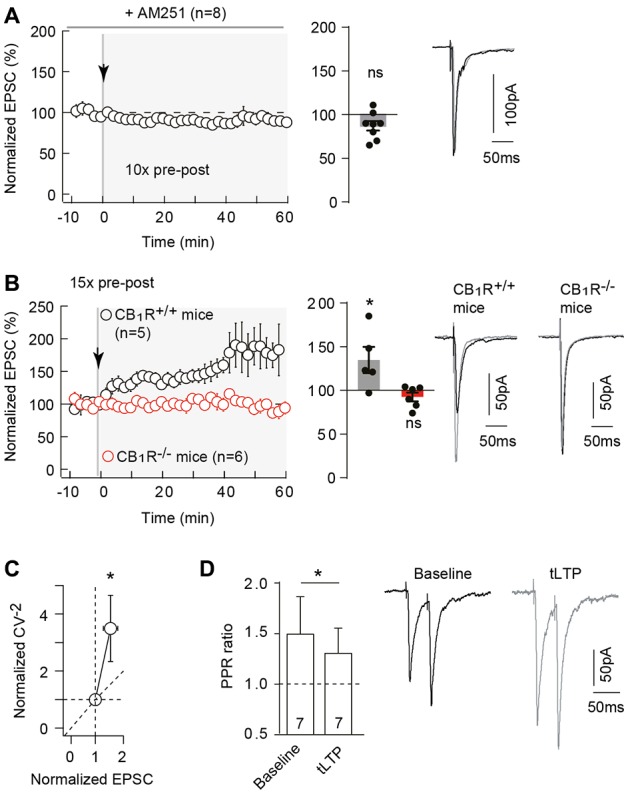
Neocortical 10 pairings-tLTP is type-1 cannabinoid receptor (CB_1_R)-mediated. **(A)** Pharmacological inhibition of CB_1_R prevented 10 pairings-tLTP. Summary of experiments showing that tLTP expression was prevented by AM251 (*n* = 8 cells from 4 rats; Δt_STDP_ = +13 ± 1 ms). **(B)** tLTP was absent in CB_1_R^−/–^ mice. Summary of experiments showing that 15 pre-post pairings induced tLTP in CB_1_R^+/+^ mice (*n* = 5 cells from 3 mice; Δt_STDP_ = +10 ± 1 ms) whereas no plasticity was observed in CB_1_R^−/–^ littermate mice (*n* = 6 cells from 5 mice; Δt_STDP_ = +10 ± 1 ms). **(C)** The mean variance analysis, to determine the plasticity locus, consists in plotting the normalized CV^−2^ (CV^−2^ after plasticity induction /CV^−2^ during baseline) with the normalized EPSC amplitude (EPSC amplitude after plasticity induction /EPSC amplitude during baseline; see “Materials and Methods” section). Normalized CV^−2^ ≥ normalized EPSCs amplitude (*n* = 17) indicates a presynaptic locus of the endocannabinoid-mediated spike-timing dependent LTP (eCB-tLTP). **(D)** Representative traces and summary bar graphs (*n* = 7 pyramidal cells) of paired-pulse stimulation with 50 ms interstimulus interval illustrate a decrease of facilitation after STDP. This suggests a presynaptic locus of the eCB-tLTP. Representative traces are the average of 15 EPSCs during baseline (black traces) and 50 min after STDP protocol (gray traces). Error bars represent SD. *p < 0.05. ns, not significant.

Pharmacological inhibition of CB_1_R with AM251 (3 μM) prevented the induction of 10 pairings-induced tLTP as illustrated in the representative experiment (Supplementary Figure S3A) and in the summary graph (87.3 ± 5.4%, *p* = 0.0511, *n* = 8; 0/8 cells displayed tLTP; Figure 4A). Note that AM251 had no effect on synaptic transmission (normalized EPSC_(baseline-drugs/baseline-control)_ amplitude: 92.4 ± 6.6%, *p* = 0.3337, *n* = 5). This pharmacological observation was further confirmed by the use of CB_1_R-knockout (CB_1_R^−/–^) mice (Ledent et al., 1999). We first ensured that we could observe tLTP induced with a few number of pre-post pairings (*N*_pairings_ = 15 in C57BL/6 mice; see “Materials and Methods” section) in littermate wild-type (CB_1_R^+/+^) mice as shown in the representative experiment (Supplementary Figure S3B1) and in the summary graph (134.8 ± 14.9%, *p* = 0.0480, *n* = 5; 4/5 cells displayed tLTP; Figure 4B). In CB_1_R^−/–^ mice, no significant plasticity was detected after 15 pre-post pairings, as shown in the example in the Supplementary Figure S3B2. To summarize, no significant plasticity was observed following 15 pre-post pairings (92.5 ± 5.0% of the baseline, *p* = 0.1948, *n* = 6; 0/6 cells displayed tLTP; Figure 4B). Pharmacological and genetic evidence indicated that tLTP induced by 10–15 pre-post pairings is CB_1_R-mediated and therefore eCB-dependent. We refer to this new form of cortical tLTP as eCB-tLTP.

CB_1_Rs are mainly located on presynaptic terminals (Katona and Freund, 2012), the locus of eCB-tLTP is thus expected to be presynaptic. To test this, we used the mean variance analysis of EPSCs (Clements and Silver, 2000). The coefficients of variation (CV) of EPSC amplitude were estimated during baseline and after plasticity induction (40 min after pairing protocol). The normalized CV^−2^ (CV^−2^ after plasticity induction /CV^−2^ during baseline) is plotted with the normalized EPSC amplitude; see “Materials and Methods” section). We obtained a CV^−2^ value of 3.5 ± 1.2 (*p* = 0.0048, *n* = 17) associated with a change in normalized EPSC amplitude of 1.6 ± 0.2 (*p* = 0.0063). Normalized CV^−2^ ≥ normalized EPSC amplitude, indicated a presynaptic locus for eCB-tLTP (Figure 4C). This was confirmed by applying paired pulses with 50 ms interpulse interval (which induced a significant EPSC paired-pulse facilitation, PPF) before and after STDP protocol (Figure 4D). EPSC facilitation was 149.6 ± 37.1% (*p* = 0.0210) and 130.6 ± 24.8% (*p* = 0.0699) before and after STDP pairings (*n* = 7), respectively. We observed a significant decrease of PPF (PPF_plasticity/baseline_ = 0.923 ± 0.046, *p* = 0.040, *n* = 7) indicating a presynaptic locus of the plasticity downstream of CB_1_Rs.

**Figure 5:**
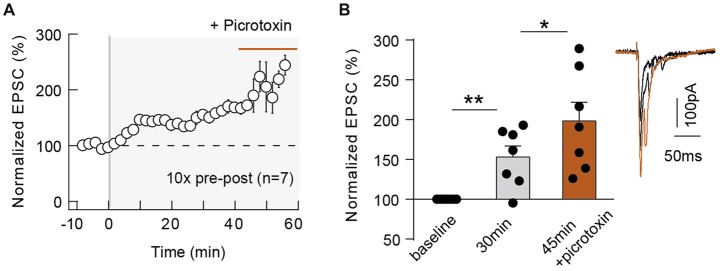
GABAergic circuits control the magnitude of 10 pairings-eCB-tLTP. Summary of the time course of tLTP induced by 10 pre-post pairings in control conditions (until 40 min after pairings) and during picrotoxin application (from 40 until 60 min; *n* = 7 cells from 4 rats; Δt_STDP_ = +14 ± 1 ms; **A**). Summary bar graph illustrating an increase of tLTP upon GABAergic transmission blockade **(B)**. Representative traces are the average of 15 EPSCs during baseline (black traces) and 50 min after STDP protocol (gray traces). Error bars represent SD. *p < 0.05; **p < 0.01.

The magnitude of neocortical eCB-tLTP could be affected by a decrease of the GABA release, via an activation of CB_1_Rs located on GABAergic terminals. Indeed, eCB-induced depression of GABAergic transmission leading to a facilitation of LTP magnitude has been observed in the hippocampus (Carlson et al., 2002; Chevaleyre and Castillo, 2004; Zhu and Lovinger, 2007; Lin et al., 2011; Xu et al., 2012). To this aim, we blocked the GABA_A_ receptors with bath-applied picrotoxin (50 μM) after STDP induction. First, we tested whether picrotoxin affects EPSC transmission during baseline and found no significant variation of EPSC amplitude after picrotoxin application (normalized EPSC_(baseline−drugs/baseline−control)_ amplitude: 105.3 ± 7.0, *p* = 0.6358, *n* = 8). Forty minutes after pairings; with 10 pre-post pairings, tLTP was still observed as shown with the example in the Supplementary Figure S3C: the mean baseline EPSC amplitude was 136 ± 32 pA before pairings, was increased by 93% to 263 ± 37 pA 40 min after pairings and was further increased by 116% (compared with baseline) to 294 ± 32 pA after picrotoxin application (1 h after pairings). In summary, picrotoxin did not impair tLTP (153.1 ± 13.9%, *p* = 0.0089, *n* = 7, before picrotoxin and 198.3 ± 13.8%, *p* = 0.0004, *n* = 7; 7/7 cells displayed tLTP) but induced an increase in the tLTP magnitude (*p* = 0.0350; Figure 5), illustrating that GABAergic microcircuits exert an inhibitory brake on eCB-tLTP.

### Neocortical eCB-tLTP Relies on TRPV1 Activation

**Figure 6:**
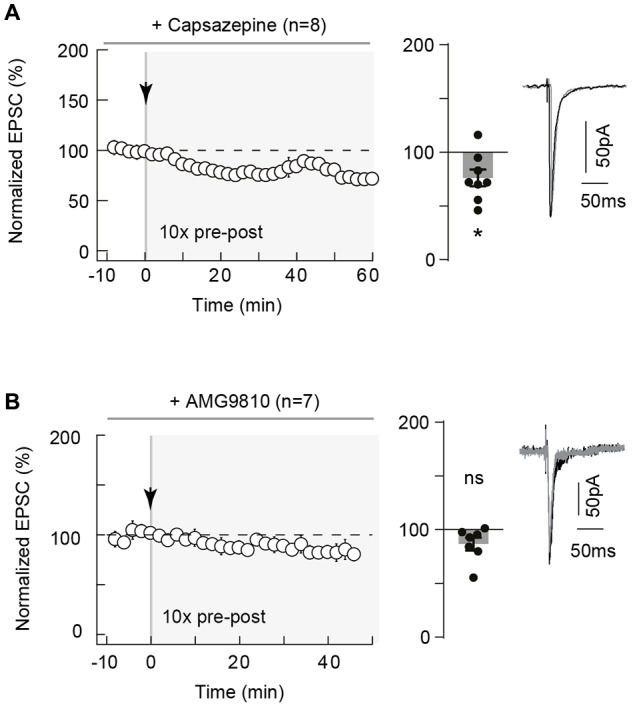
Neocortical eCB-tLTP relies on transient receptor potential vanilloid type-1 (TRPV1) activation. **(A)** 10 pairings-tLTP was prevented when TRPV1 was inhibited by capsazepine, a specific TRPV1 inhibitor. Summary of experiments showing that tLTP expression was prevented by capsazepine (*n* = 8 cells from 4 rats; Δt_STDP_ = +17 ± 2 ms). **(B)** 10 pairings-tLTP was prevented when TRPV1 was inhibited by AMG9810, a specific TRPV1 inhibitor. Summary of experiments showing that AMG9810 prevented the 10 pairings tLTP (*n* = 7 cells from 3 rats; Δt_STDP_ = +16 ± 1 ms). Representative traces are the average of 15 EPSCs during baseline (black traces) and 50 min after STDP protocol (gray traces). Error bars represent SD. *p < 0.05. ns, non significant.

Neuronal activity can lead to the synthesis and release of various eCBs, which includes 2-AG but also anandamide (Piomelli et al., 2007; Alger and Kim, 2011). Whereas 2-AG is a specific ligand of CB_1_R, anandamide activates not only CB_1_R (less potently than 2-AG) but also TRPV1. TRPV1 is a cationic channel highly permeable to calcium and is activated by anandamide, one of the major eCB with 2-AG (Starowicz et al., 2007; Di Marzo, 2008), which has been reported to be involved in eCB-mediated short- and long-term depression in hippocampus (Gibson et al., 2008; Chávez et al., 2010), in superior colliculus (Maione et al., 2009) and in the extended amygdala (Grueter et al., 2010; Puente et al., 2011) but also in eCB-tLTP in dorsolateral striatum (Cui et al., 2015). We, therefore, investigated the involvement of TRPV1 in neocortical eCB-tLTP. We first assessed the presence of functional TRPV1 at neocortical synapses by applying capsaicin (10 μM), a TRPV1 agonist, and we observed that EPSCs were decreased (79.1 ± 6.2% of baseline, *p* < 0.05, *n* = 5; data not shown). This is in in agreement with the expression of TRPV1 in pyramidal cells of the rat neocortex revealed by immunoelectronmicroscopy (Tóth et al., 2005), real-time PCR (Huang et al., 2014; but see Cavanaugh et al., 2011) or functional (Pezzoli et al., 2014) analysis. We next used capsazepine, a competitive TRPV1 antagonist, and first verified that capsazepine (10 μM) had no significant effect on basal EPSCs (105.2 ± 5.5%, *p* = 0.3980, *n* = 5) in absence of paired stimulation, indicating that TRPV1 has no constitutive activity at neocortical layer 2/3 synapses. Capsazepine (10 μM) bath-application during STDP (10 pre-post pairings) prevented tLTP expression as illustrated in the example in the Supplementary Figure S3D and in the summary graph (76.2 ± 7.8%, *p* = 0.0184, *n* = 8; 0/8 cells displayed tLTP; Figure 6A). To confirm this finding, we next used another competitive TRPV1 antagonist, AMG9810, structurally distinct from capsazepine. AMG9810 (1 μM) had no effect on basal synaptic transmission (normalized EPSC_(baseline-drugs/baseline-control)_ amplitude: 105.2 ± 6.6%, *p* = 0.4748, *n* = 5). In the example in the Supplementary Figure S3E, 10 pre-post pairings with bath-applied AMG9810 failed to induce tLTP. In summary, we observed that AMG9810 prevented eCB-tLTP (86.6 ± 5.9%, *p* = 0.0649, *n* = 7; 0/7 cells displayed tLTP; Figure 6B).

Altogether, our results demonstrate that 10 pairings-tLTP is mediated by both CB_1_R and TRPV1 activation.

## Discussion

In rodent neocortex, we report here the existence of a Hebbian coincidence-activity dependent LTP induced by a low number of pairings (~10), which involved the eCB system. eCB-tLTP induction relies on activation of CB_1_R and TRPV1 triggered by coupled rises of calcium mediated by mGluR5 (via PLCß, diacylglycerol lipase-α activation and calcium released from internal stores) and VSCCs. Most of the steps of eCB synthesis and release (mainly 2-AG and anandamide) tightly depend on postsynaptic calcium levels (and time course; Piomelli et al., 2007; Di Marzo, 2008; Kano et al., 2009; Alger and Kim, 2011). Due to their on-demand intercellular signaling (Piomelli et al., 2007; Alger and Kim, 2011), eCB action is expected to be controlled by precisely timed stimuli. Here we show that STDP, a Hebbian synaptic learning rule (Dan and Poo, 2006; Sjöström et al., 2008; Feldman, 2012; Froemke, 2015), efficiently triggers eCB signaling, even for a low number of pairings, and can promote the expression of eCB-tLTP. Therefore, in addition, to the widespread eCB-LTD, eCBs can aslo mediate potentiation. Bidirectionality of eCB-plasticity is a crucial property of eCB system because it allows eCB-LTP and -LTD to reverse each other at a single synapse. We have previously proposed a mechanism accounting for corticostriatal eCB-tLTP using a combination of patch-clamp recordings and a mathematical model (Cui et al., 2016). In our model, low to moderate peak levels of eCB would lead to tLTD whereas high eCB levels would yield tLTP. It is thus expected that the first 10 pairings produce large peak levels of eCB synthesis, thus inducing tLTP. If the amplitude of the 2-AG peaks decreases for subsequent pairings, this initial tLTP would be de-potentiated; its expression would be thus restricted for few coincident activity and eCB would have no significant impact on tLTP induced with 100 pairings. Supporting this, it has been reported that in the somatosensory cortex NMDAR-tLTP expression (induced with 100 pre-post pairings) was not modified after the inhibition of CB_1_Rs (Bender V. A. et al., 2006; Nevian and Sakmann, 2006).

We observed eCB-tLTP in both somatosensory cortex and in dorsolateral striatum, demonstrating that this form of plasticity is not restricted to a single type of synapses, but could serve as a general system in various brain structures to allow the engram of salient events from few spikes. Neocortical eCB-tLTP is similar to those we recently described in the dorsal striatum (Cui et al., 2015, 2016) with the noticeable difference that this new form of plasticity displays, a distinct polarity depending on its synaptic site of expression: Hebbian in neocortex and anti-Hebbian in striatum. Interestingly, eCB-tLTP only requires a single postsynaptic spike per paired stimulation in the dorsal striatum but 2–3 in the neocortex, possibly consistent with the general observation that learning dynamics are usually faster in sub-cortical structures than in the neocortex (Pasupathy and Miller, 2005). It remains to be investigated whether neuromodulators control eCB-tLTP expression and/or polarity, as reported for dopamine for the control of the NMDAR-tLTP in prefrontal or visual cortex (Seol et al., 2007; Xu and Yao, 2010). Furthermore, it has been reported that in a STDP paradigm noradrenaline and serotonin transform eligibility traces into plasticity in the visual cortex (He et al., 2015). In the same line, it is important to explore whether neuromodulators could promote the emergence of eCB-tLTP for lower number of pairings or with a single backpropagating action potential (instead of 2–3 as it is the case in the present study).

eCB system is deeply involved in learning and memory (Mechoulam and Parker, 2013) because its major role in synaptic plasticity expression (Augustin and Lovinger, 2018). Indeed, eCBs (2-AG and anandamide) have been extensively reported to mediate short- or long-term depression, via the activation of CB_1_R (Kano et al., 2009; Castillo et al., 2012; Katona and Freund, 2012; Melis et al., 2014) or TRPV1 (Gibson et al., 2008; Maione et al., 2009; Chávez et al., 2010; Grueter et al., 2010; Puente et al., 2011). Nevertheless, it exists now as a growing body of evidence showing an indirect role of eCBs in promoting short- or LTP (reviewed in: Araque et al., 2017; Augustin and Lovinger, 2018): at mixed synapses of the goldfish Mauthner cell *via* intermediary dopaminergic neurons (Cachope et al., 2007) or at CA1 synapses in hippocampus via a GABA_A_ receptor-mediated mechanism (Lin et al., 2011; Xu et al., 2012). It has also been reported in the hippocampus that eCB-induced presynaptic depression of GABAergic transmission facilitates LTP (Carlson et al., 2002; Chevaleyre and Castillo, 2004; Zhu and Lovinger, 2007), or that eCBs mediate heterosynaptic short-term potentiation *via* intermediary astrocytes (Navarrete and Araque, 2010). More recently, it has been reported a direct role of eCBs in promoting LTP at cortical inputs to the granule cells of the dentate gyrus (Wang et al., 2016, 2018), to the medium-sized spiny neurons of the dorsolateral striatum (Cui et al., 2015, 2016) or to the basal dendrites of layer 5 pyramidal cells (in this later case the eCB-LTP is also BDNF- and NMDAR-mediated; Maglio et al., 2018). At hippocampal CA1 synapses, eCB-mediated LTP induced with high-frequency (Lin et al., 2011), low-frequency (Zhu and Lovinger, 2007) or paired (Xu et al., 2012) stimulations were prevented by inhibition of CB_1_R and GABA_A_ receptors. Here, we show at neocortical synapses that GABA, which does not affect EPSC amplitude during baseline, controls the plasticity magnitude by exerting a brake on eCB-tLTP. In line with previous studies showing the existence of eCB-LTP at striatal (Cui et al., 2015, 2016), hippocampal (Wang et al., 2016, 2018) and cortical (Maglio et al., 2018) synapses, the present neocortical eCB-tLTP constitutes an example of a paired-activity eCB-LTP with a direct implication of eCBs (see i-THL experiments) in the induction of tLTP of the stimulated synapse itself. It remains to be determined whether eCB-tLTP expression could be extended to other brain structures and synapses.

Evidence for TRPV1 activation by physiological neuronal activity remains unclear. In rodent neocortex, it is fair to say that the expression of TRPV1 in pyramidal cells was debated. Indeed, although an immunoelectronmicroscopy study reported TRPV1 protein in neocortex at the postsynaptic dendritic spines of pyramidal cells (Tóth et al., 2005), a multi-approach investigation (*in situ* hybridization, calcium imaging) detects TRPV1 only at the level of the arteriolar smooth muscle cells (Cavanaugh et al., 2011). Recent studies using real-time PCR and western blot (Huang et al., 2014) and patch-clamp recordings (Pezzoli et al., 2014) showed expression of TRPV1 by pyramidal cells of the neocortex. Here, EPSC recordings, before and after TRPV1 agonist (capsaicin) application, show a marked decrease of EPSC amplitude in presence of capsaicin, in line with previous observations (Pezzoli et al., 2014). In addition, we found that two competitive TRPV1 antagonists prevent eCB-tLTP expression. As previously described in striatum (Cui et al., 2015), our study confirms that STDP efficiently triggers eCB signaling and is able to recruit the TRPV1 signaling pathway. TRPV1 is a cationic channel highly permeable to calcium (Starowicz et al., 2007; Di Marzo, 2008) and may contribute to eCB-tLTP induction by boosting the calcium transients. Here, TRPV1 is most likely activated by anandamide arising from the stimulated postsynaptic cell. In the dorsolateral striatum, anandamide appears necessary but not sufficient for tLTP induction (Cui et al., 2016). In the neocortex, it remains to be determined whether anandamide alone could trigger eCB-tLTP induction following limited occurrences of coincident activity. As described for eCB-mediated LTD (Puente et al., 2011), our results illustrate the bidirectionality of eCBs as a system exhibiting polymodal activation through CB_1_R and TRPV1, to induce LTD and LTP.

The neocortex receives a broad range of cortical activity patterns, from isolated trains of few spikes to sustained bursting events. At neocortical layer 4-2/3 synapses, STDP exhibits symmetric Hebbian tLTP during the first two postnatal weeks (Itami and Kimura, 2012), then asymmetric Hebbian STDP (Feldman, 2000; Sjöström et al., 2001, 2003; Bender K. J. et al., 2006; Bender V. A. et al., 2006; Froemke et al., 2006; Nevian and Sakmann, 2006; Banerjee et al., 2014); a lack of tLTD has been reported for older animals (Banerjee et al., 2009 but see Min and Nevian, 2012). It should be noted that depending on the site of stimulation (layer 4 or at within layer 2/3), the temporal window and the locus of NMDAR involved of tLTD are different: for post-pre pairings, stimulation in layer 4 induced a tLTD dependent on presynaptic NMDAR which is expressed in a broad Δt_STDP_, whereas stimulation within layer 2/3 triggered a tLTD dependent on postsynaptic NMDAR which is expressed in a more restricted Δt_STDP_ (Banerjee et al., 2014). Although cortical plasticity under prolonged activation (low- and high-frequency stimulations, theta bursts or 100 pairings STDP) is well elucidated, its expression in response to few spikes remained elusive. Nevertheless, it has been observed that dendritic spike(s) induced by single-shock and single-burst were responsible for, respectively, LTD in visual cortex (Holthoff et al., 2004) and LTP in hippocampus (Remy and Spruston, 2007). These both plasticity was NMDAR-mediated and could thus account for single-trial learning. eCB-LTP is promoted by about 10 of pairings, allowing for the synapses to react to the first occurrences of incoming activity. In the same line, ~20–25 STDP pairings induced LTP in hippocampal neurons (Zhang et al., 2009) or at layer 2/3 cortical pyramidal cells (Froemke et al., 2006); interestingly, in hippocampal neurons bath-application of dopamine allows the induction of tLTP with a lower number of pairings (10 instead of 20 pairings; Zhang et al., 2009). Associative memories and behavioral rules can be learned with few trials (5–10) or even with one trial (Schultz et al., 2003; Pasupathy and Miller, 2005; Armstrong et al., 2006; Rutishauser et al., 2006; Whitlock et al., 2006; Tse et al., 2007; Quilodran et al., 2008; Cook and Fagot, 2009; Ito and Doya, 2009; Izquierdo et al., 2016). Upon behaviorally pertinent events, neurons with behavior-related activities fire a few spikes during each trial (one to a dozen) upon each trial (i.e., a discharge at frequency 5–10 Hz during 0.1–0.5 s; Schultz et al., 2003; Pasupathy and Miller, 2005; Quilodran et al., 2008). This suggests that a low number (2–50) of spikes should be sufficient for the expression of synaptic plasticity. The present results suggest that eCB-tLTP could be involved for learning salient events from a low number of action potentials and may constitute a neuronal substrate for single-trial or online learning, such as cortical episodic memory. It remains to be investigated if eCB-tLTP occurs *in vivo* and to evaluate the involvement of eCB-tLTP in the initial phases of online learning, which could be thereafter reinforced by NMDAR-LTP when stimuli are subsequently repeated.

Frequent cannabis use leads to impairment of working memory in the left superior parietal cortex (Jager et al., 2006) as well as long-term memory via activation of CB_1_R (Mechoulam and Parker, 2013; Augustin and Lovinger, 2018). This impairment was mainly interpreted as the effect of cannabinoids on the induction of short- and long-term synaptic depression. Our results, in line with other studies (Lin et al., 2011; Xu et al., 2012; Cui et al., 2015; Wang et al., 2016; Maglio et al., 2018), indicate that potentiation of synaptic transmission may also be involved in the effects of marijuana intoxication.

## Author Contributions

LV, YC and SP: conception and design of the experiments. YC and SP performed electrophysiological experiments. YC, SP and LV performed data analysis. LV: writing of original draft with contributions from YC.

## Conflict of Interest Statement

The authors declare that the research was conducted in the absence of any commercial or financial relationships that could be construed as a potential conflict of interest.

## Supplementary Material

FIGURE S1 Representative STDP experiments related to Figure 1. **(A)** 100 pairings induced bidirectional Hebbian plasticity. **(A1)** Example of tLTD induced by 100 post-pre pairings (Δt_STDP_ = −13 ms). EPSC strength before and after pairings (before pairings: 198 ± 3 pA; 45–55 min after pairings: 106 ± 2 pA; decrease of 46%). Bottom, time courses of Ri (before, 224 ± 4 MΩ; after, 252 ± 2 MΩ; change of 12%) and Iinj (before, 13 ± 1 pA; after, 34 ± 1 pA) for this cell. **(A2)** Example of tLTP induced by 100 pre-post pairings (Δt_STDP_ = +10 ms). EPSC strength before and after pairings (before pairings: 72 ± 2 pA; 45–55 min after pairings: 126 ± 2 pA; increase of 74%). Bottom, time courses of Ri (before, 285 ± 3 MΩ; after, 297 ± 2 MΩ; change of 4%) and Iinj (before, 5 ± 0.2 pA; after, 3 ± 0.3 pA). **(B)** 50 pairings induced symmetric anti-Hebbian tLTD. **(B1)** Example of tLTD induced by 50 post-pre pairings (Δt_STDP_ = −15 ms). EPSC strength before and after pairings (before pairings: 131 ± 2 pA; 45–55 min after pairings: 92 ± 2 pA; decrease of 29%). Bottom, time courses of Ri (before, 139 ± 1 MΩ; after, 136 ± 1 MΩ) and Iinj (before, 13 ± 1 pA; after, 34 ± 1 pA). **(B2)** Example of tLTD induced by 50 pre-post pairings (Δt_STDP_ = +14 ms). EPSC strength before and after pairings (before pairings: 266 ± 5 pA; 45–55 min after pairings: 133 ± 3 pA; decrease of 50%). Bottom, time courses of Ri (before, 151 ± 1 MΩ; after, 141 ± 1 MΩ; change of 6%) and Iinj (before, 16 ± 1 pA; after, −10 ± 1 pA). **(C)** 10 pairings induced unidirectional Hebbian plasticity. **(C1)** Example of a lack of plasticity induced by 10 post-pre pairings (Δt_STDP_ = −13 ms). EPSC strength before and after pairings show no significant change (before pairings: 138 ± 23 pA; 45–55 min after pairings: 149 ± 20 pA; variation of 8%). Bottom, time courses of Ri (before, 204 ± 2 MΩ; after, 201 ± 1 MΩ; change of 1.5%) and Iinj (before, −5 ± 0.3 pA; after, −5 ± 0.5 pA). **(C2)** Example of tLTP induced by 10 pre-post pairings (Δt_STDP_ = +17 ms). EPSC strength before and after pairings (before pairings: 45 ± 2 pA; 45–55 min after pairings: 81 ± 2 pA; increase of 79%). Bottom, time courses of Ri (before, 388 ± 6 MΩ; after, 458 ± 6 MΩ; change of 18%) and Iinj (before, 10 ± 0.4 pA; after, 3 ± 0.3 pA). **(D)** Relationship between the synaptic efficacy changes after 10 pre-post pairings and the EPSC amplitude during the baseline (10 pre-post pairings, *R*^2^ = 0.1012, *p* = 0.2478, *n* = 15). Representative traces are the average of 15 EPSCs during baseline (black traces) and 50 min after STDP protocol (gray traces). Supplementary Figure S1 is related to Figure 1.

FIGURE S2 Representative STDP experiments related to Figures 2, 3. **(A)** Example of tLTP induced by 10 pre-post pairings (Δt_STDP_ = +8 ms) in presence of D-AP5 (50 μM). EPSC strength before and after pairings (before pairings: 70 ± 1 pA; 45–55 min after pairings: 106 ± 2 pA; increase of 50%). Bottom, time courses of Ri (before, 274 ± 32 MΩ; after, 299 ± 19 MΩ; change of 9%) and Iinj (before, 18 ± 10 pA; after, −7 ± 4 pA) for this cell. **(B)** Example of an inhibition of tLTP in presence of MPEP (10 μM), an antagonist of mGluR5, with 10 pre-post pairings (Δt_STDP_ = +12 ms). EPSC strength before and after pairings show no significant change (before pairings: 292 ± 20 pA; 45–55 min after pairings: 277 ± 24 pA; variation of 5%). Bottom, time courses of Ri (before, 124 ± 1 MΩ; after, 104 ± 1 MΩ; change of 16%) and Iinj (before, 39 ± 4 pA; after, −30 ± 3 pA). **(C)** Example showing that nimodipine (1 μM) prevented tLTP, with 10 pre-post pairings (Δt_STDP_ = +10 ms). EPSC strength before and after pairings show no significant change (before pairings: 293 ± 39 pA; 45–55 min after pairings: 278 ± 29 pA; variation of 5%). Bottom, time courses of Ri (before, 324 ± 3 MΩ; after, 316 ± 5 MΩ; change of 2%) and Iinj (before, −3 ± 0.4 pA; after, −36 ± 0.5 pA). **(D)** Example showing that THL, applied intracellularly via the patch pipette (i-THL, 10 μM) prevented tLTP, with 10 pre-post pairings. EPSC strength before and after pairings show no significant change (before pairings: 155 ± 3 pA; 45–55 min after pairings: 84 ± 2 pA; variation of 46%). Bottom, time courses of Ri (before, 178 ± 3 MΩ; after, 184 ± 2 MΩ; change of 3%) and Iinj (before, −18 ± 2 pA; after, −31 ± 1 pA). Representative traces are the average of 15 EPSCs during baseline (black traces) and 50 min after STDP protocol (gray traces).

FIGURE S3 Representative STDP experiments related to Figures 4–6. **(A)** Example showing that a specific CB_1_R inhibitor, AM251 (3 μM), prevented tLTP; STDP pairings consisted in 10 pre-post pairings (Δt_STDP_ = +14 ms). EPSC strength before and after pairings shows no significant change (before pairings: 174 ± 23 pA; 45–55 min after pairings: 171 ± 31 pA; variation of 1%). Bottom, time courses of Ri (before, 230 ± 23 MΩ; after, 270 ± 33 MΩ; change of 17%) and Iinj (before, 1 ± 1 pA; after, 11 ± 0.3 pA). **(B1)** Example of tLTP induced by 15 pre-post pairings (Δt_STDP_ = +13 ms) in wild-type CB_1_R^+/+^ mice. EPSC strength before and after pairings (before pairings: 88 ± 18 pA; 45–55 min after pairings: 170 ± 29 pA; increase of 93%). Bottom, time courses of Ri (before, 194 ± 3 MΩ; after, 211 ± 2 MΩ; change of 9%) and Iinj (before, 5 ± 1 pA; after, 8 ± 1 pA). **(B2)** Representative experiment showing that 15 pre-post pairings (Δt_STDP_ = +14 ms) failed to induce tLTP in CB_1_R^−/–^ mice. EPSC strength before and after pairings (before pairings: 155 ± 24 pA; 45–55 min after pairings: 137 ± 28 pA; variation of 12%). Bottom, time courses of Ri (before, 130 ± 1 MΩ; after, 139 ± 1 MΩ; change of 1%) and Iinj (before, −22 ± 2 pA; after, −7 ± 0.3 pA). **(C)** Example of tLTP induced by 10 pre-post pairings (Δt_STDP_ = +14 ms) with picrotoxin (50 μM), a blocker of ionotropic GABAergic transmission. EPSC strength before and after pairings (before pairings: 136 ± 32 pA; 35–40 min after pairings 263 ± 37 pA; increase of 93% after picrotoxin treatment 40 min after pairings 294 ± 32 pA; increase of 116%). Bottom, time courses of Ri (before, 217 ± 3 MΩ; after, 205 ± 1 MΩ; change of 6%) and Iinj (before, −11 ± 1 pA; after, −10 ± 0.2 pA) for this cell. **(D)** Representative experiment showing that 10 pre-post pairings (Δt_STDP_ = +15 ms) failed to induce tLTP with bath-applied capsazepine (10 μM). EPSC strength before and after pairings (before pairings: 185 ± 33 pA; 45–55 min after pairings: 164 ± 17 pA; variation of 11%). Bottom, time courses of Ri (before, 163 ± 1 MΩ; after, 180 ± 2 MΩ; change of 10%) and Iinj (before, 15 ± 0.5 pA; after, 29 ± 0.5 pA). **(E)** Representative experiment showing that 10 pre-post pairings (Δt_STDP_ = +14 ms) failed to induce tLTP with bath-applied AMG9810 (1 μM). Top, EPSC strength before and after pairings (before pairings: 202 ± 29 pA; 45–55 min after pairings: 162 ± 9 pA; variation of 18%). Bottom, time courses of Ri (before, 230 ± 3 MΩ; after, 201 ± 2 MΩ; change of 13%) and Iinj (before, 11 ± 2 pA; after, 3 ± 0.2 pA). Representative traces are the average of 15 EPSCs during baseline (black traces) and 50 min after STDP protocol (gray traces).

TABLE S1 Absolute values of post-pre and pre-post Δt_STDP_.

## Abbreviations

Δt_STDP_: STDP time interval
CB1R: type-1 cannabinoid receptor
eCB: endocannabinoid
mGluR5: type-5 metabotropic glutamate receptor
PLC: phospholipase
STDP: spike-timing dependent plasticity
tLTP: spike-timing dependent LTP
TRPV1: transient receptor potential vanilloid type-1
VSCCs: voltage-sensitive calcium channels.
