# Supplementary figures and images for: Endocannabinoid-LTP Mediated by CB1 and TRPV1 Receptors Encodes for Limited Occurrences of Coincident Activity in Neocortex
Source: Front Cell Neurosci. 2018 Jul 5;12:182. doi: 10.3389/fncel.2018.00182 (PMC6041431; doi:10.3389/fncel.2018.00182)

Supplementary Figure 1

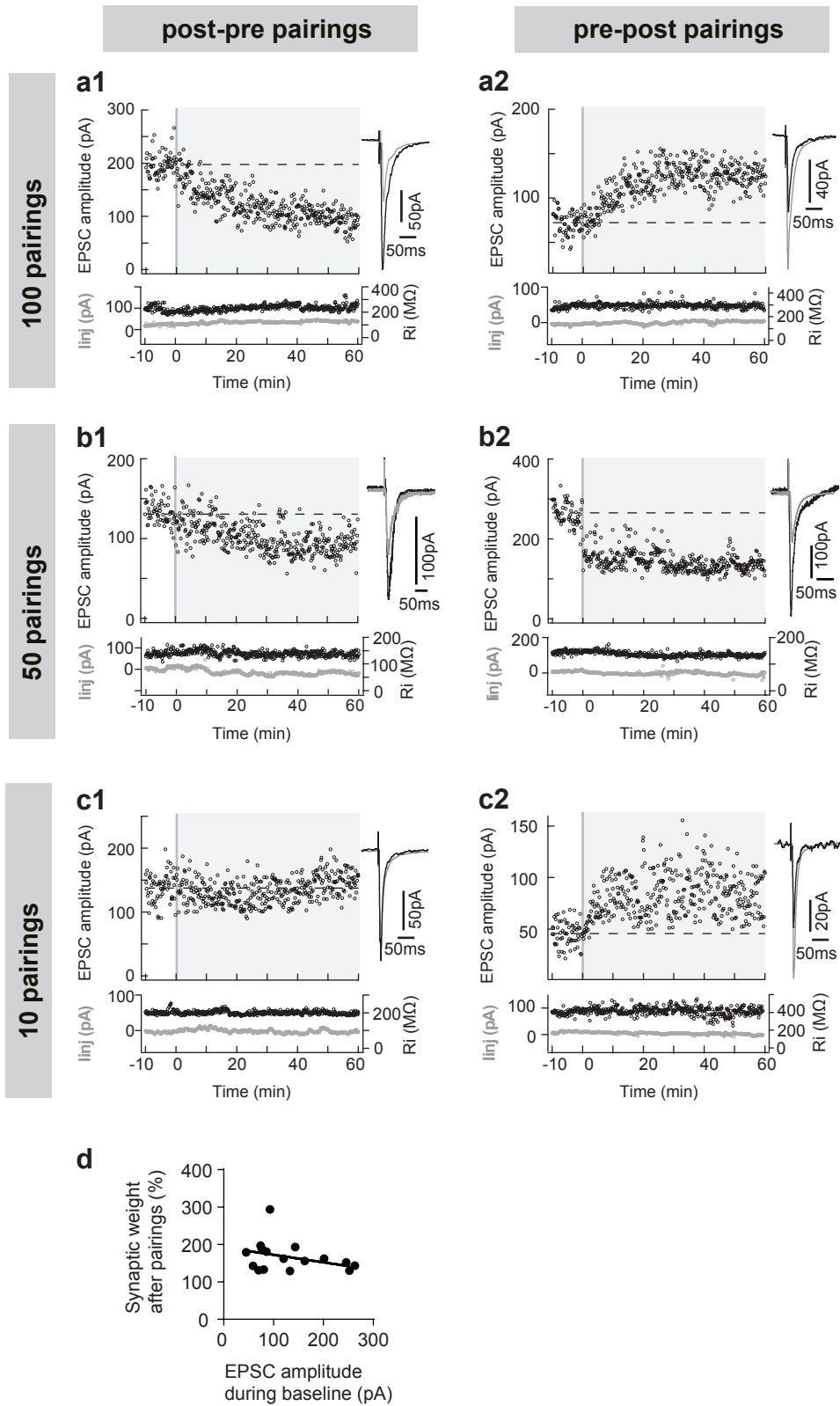

Supplement: FIGURE S1 — Representative STDP experiments related to Figure 1. (A) 100 pairings induced bidirectional Hebbian plasticity. (A1) Example of tLTD induced by 100 post-pre pairings (ΔtSTDP = −13 ms). EPSC strength before and after pairings (before pairings: 198 ± 3 pA; 45–55 min after pairings: 106 ± 2 pA; decrease of 46%). Bottom, time courses of Ri (before, 224 ± 4 MΩ; after, 252 ± 2 MΩ; change of 12%) and Iinj (before, 13 ± 1 pA; after, 34 ± 1 pA) for this cell. (A2) Example of tLTP induced by 100 pre-post pairings (ΔtSTDP = +10 ms). EPSC strength before and after pairings (before pairings: 72 ± 2 pA; 45–55 min after pairings: 126 ± 2 pA; increase of 74%). Bottom, time courses of Ri (before, 285 ± 3 MΩ; after, 297 ± 2 MΩ; change of 4%) and Iinj (before, 5 ± 0.2 pA; after, 3 ± 0.3 pA). (B) 50 pairings induced symmetric anti-Hebbian tLTD. (B1) Example of tLTD induced by 50 post-pre pairings (ΔtSTDP = −15 ms). EPSC strength before and after pairings (before pairings: 131 ± 2 pA; 45–55 min after pairings: 92 ± 2 pA; decrease of 29%). Bottom, time courses of Ri (before, 139 ± 1 MΩ; after, 136 ± 1 MΩ) and Iinj (before, 13 ± 1 pA; after, 34 ± 1 pA). (B2) Example of tLTD induced by 50 pre-post pairings (ΔtSTDP = +14 ms). EPSC strength before and after pairings (before pairings: 266 ± 5 pA; 45–55 min after pairings: 133 ± 3 pA; decrease of 50%). Bottom, time courses of Ri (before, 151 ± 1 MΩ; after, 141 ± 1 MΩ; change of 6%) and Iinj (before, 16 ± 1 pA; after, −10 ± 1 pA). (C) 10 pairings induced unidirectional Hebbian plasticity. (C1) Example of a lack of plasticity induced by 10 post-pre pairings (ΔtSTDP = −13 ms). EPSC strength before and after pairings show no significant change (before pairings: 138 ± 23 pA; 45–55 min after pairings: 149 ± 20 pA; variation of 8%). Bottom, time courses of Ri (before, 204 ± 2 MΩ; after, 201 ± 1 MΩ; change of 1.5%) and Iinj (before, −5 ± 0.3 pA; after, −5 ± 0.5 pA). (C2) Example of tLTP induced by 10 pre-post pairings (ΔtSTDP = +17 ms). EPSC strength [file Image_1.PDF]

Supplementary Figure 2

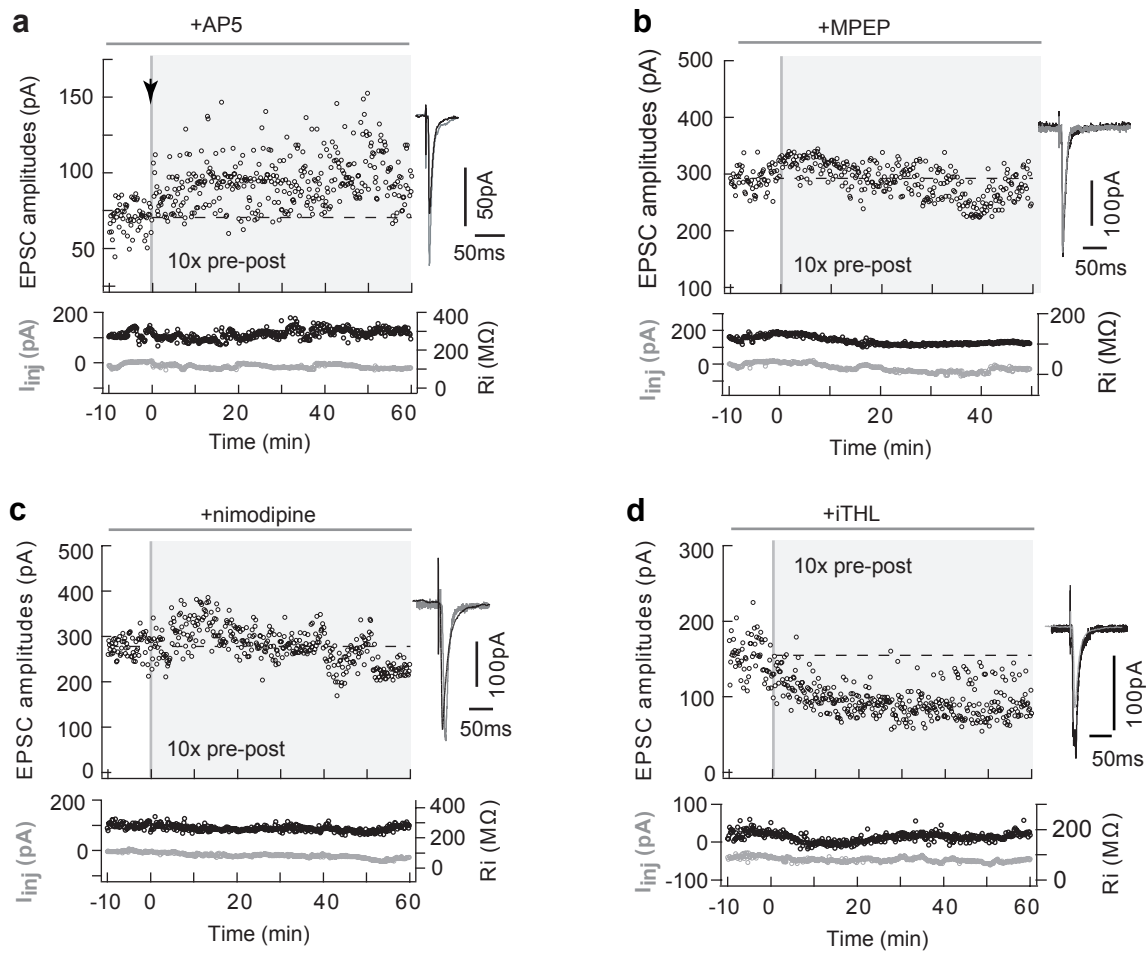

Supplement: FIGURE S2 — Representative STDP experiments related to Figures 2, 3. (A) Example of tLTP induced by 10 pre-post pairings (ΔtSTDP = +8 ms) in presence of D-AP5 (50 μM). EPSC strength before and after pairings (before pairings: 70 ± 1 pA; 45–55 min after pairings: 106 ± 2 pA; increase of 50%). Bottom, time courses of Ri (before, 274 ± 32 MΩ; after, 299 ± 19 MΩ; change of 9%) and Iinj (before, 18 ± 10 pA; after, −7 ± 4 pA) for this cell. (B) Example of an inhibition of tLTP in presence of MPEP (10 μM), an antagonist of mGluR5, with 10 pre-post pairings (ΔtSTDP = +12 ms). EPSC strength before and after pairings show no significant change (before pairings: 292 ± 20 pA; 45–55 min after pairings: 277 ± 24 pA; variation of 5%). Bottom, time courses of Ri (before, 124 ± 1 MΩ; after, 104 ± 1 MΩ; change of 16%) and Iinj (before, 39 ± 4 pA; after, −30 ± 3 pA). (C) Example showing that nimodipine (1 μM) prevented tLTP, with 10 pre-post pairings (ΔtSTDP = +10 ms). EPSC strength before and after pairings show no significant change (before pairings: 293 ± 39 pA; 45–55 min after pairings: 278 ± 29 pA; variation of 5%). Bottom, time courses of Ri (before, 324 ± 3 MΩ; after, 316 ± 5 MΩ; change of 2%) and Iinj (before, −3 ± 0.4 pA; after, −36 ± 0.5 pA). (D) Example showing that THL, applied intracellularly via the patch pipette (i-THL, 10 μM) prevented tLTP, with 10 pre-post pairings. EPSC strength before and after pairings show no significant change (before pairings: 155 ± 3 pA; 45–55 min after pairings: 84 ± 2 pA; variation of 46%). Bottom, time courses of Ri (before, 178 ± 3 MΩ; after, 184 ± 2 MΩ; change of 3%) and Iinj (before, −18 ± 2 pA; after, −31 ± 1 pA). Representative traces are the average of 15 EPSCs during baseline (black traces) and 50 min after STDP protocol (gray traces). [file Image_2.PDF]

Supplementary Figure 3

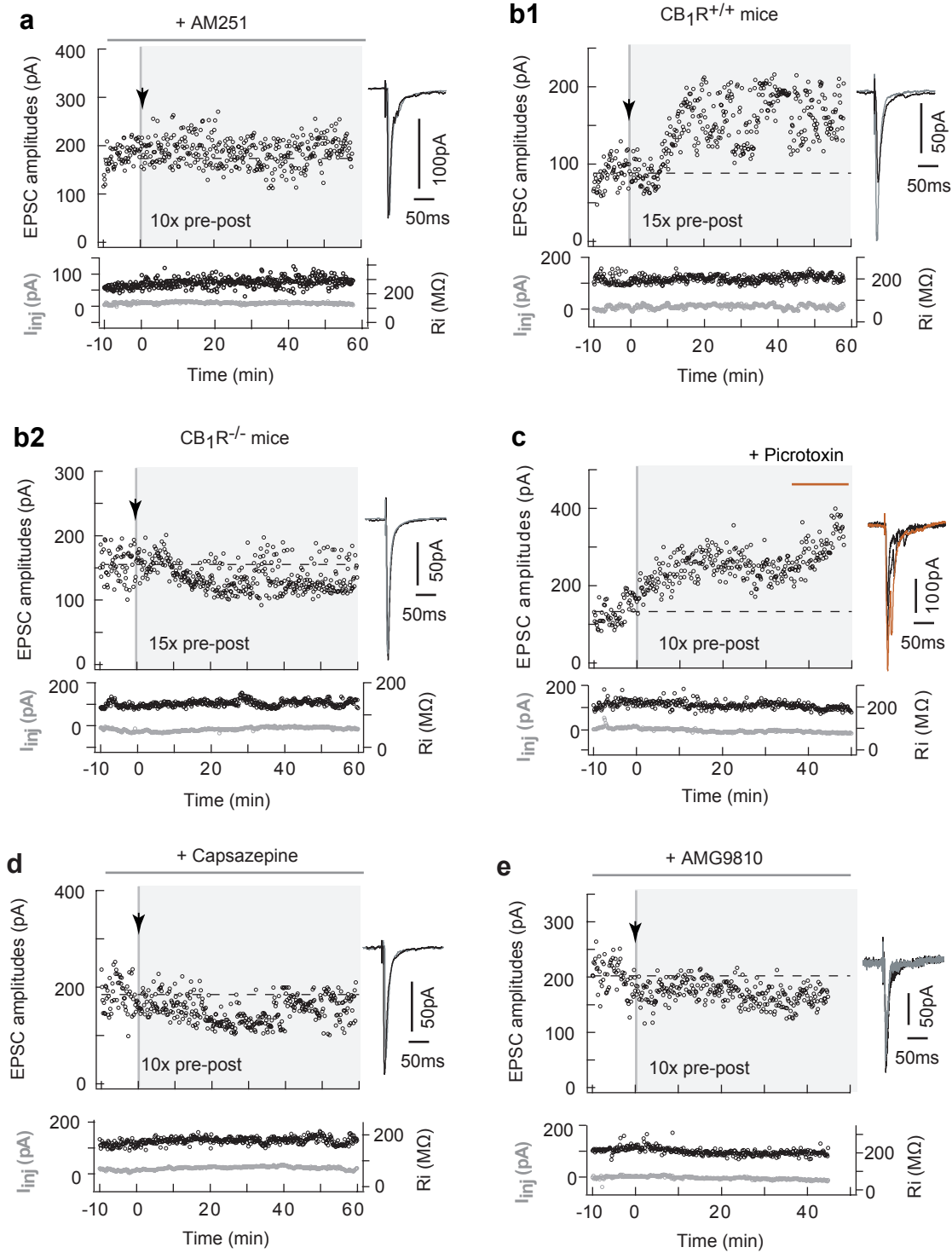

Supplement: FIGURE S3 — Representative STDP experiments related to Figures 4–6. (A) Example showing that a specific CB1R inhibitor, AM251 (3 μM), prevented tLTP; STDP pairings consisted in 10 pre-post pairings (ΔtSTDP = +14 ms). EPSC strength before and after pairings shows no significant change (before pairings: 174 ± 23 pA; 45–55 min after pairings: 171 ± 31 pA; variation of 1%). Bottom, time courses of Ri (before, 230 ± 23 MΩ; after, 270 ± 33 MΩ; change of 17%) and Iinj (before, 1 ± 1 pA; after, 11 ± 0.3 pA). (B1) Example of tLTP induced by 15 pre-post pairings (ΔtSTDP = +13 ms) in wild-type CB1R+/+ mice. EPSC strength before and after pairings (before pairings: 88 ± 18 pA; 45–55 min after pairings: 170 ± 29 pA; increase of 93%). Bottom, time courses of Ri (before, 194 ± 3 MΩ; after, 211 ± 2 MΩ; change of 9%) and Iinj (before, 5 ± 1 pA; after, 8 ± 1 pA). (B2) Representative experiment showing that 15 pre-post pairings (ΔtSTDP = +14 ms) failed to induce tLTP in CB1R−/– mice. EPSC strength before and after pairings (before pairings: 155 ± 24 pA; 45–55 min after pairings: 137 ± 28 pA; variation of 12%). Bottom, time courses of Ri (before, 130 ± 1 MΩ; after, 139 ± 1 MΩ; change of 1%) and Iinj (before, −22 ± 2 pA; after, −7 ± 0.3 pA). (C) Example of tLTP induced by 10 pre-post pairings (ΔtSTDP = +14 ms) with picrotoxin (50 μM), a blocker of ionotropic GABAergic transmission. EPSC strength before and after pairings (before pairings: 136 ± 32 pA; 35–40 min after pairings 263 ± 37 pA; increase of 93% after picrotoxin treatment 40 min after pairings 294 ± 32 pA; increase of 116%). Bottom, time courses of Ri (before, 217 ± 3 MΩ; after, 205 ± 1 MΩ; change of 6%) and Iinj (before, −11 ± 1 pA; after, −10 ± 0.2 pA) for this cell. (D) Representative experiment showing that 10 pre-post pairings (ΔtSTDP = +15 ms) failed to induce tLTP with bath-applied capsazepine (10 μM). EPSC strength before and after pairings (before pairings: 185 ± 33 pA; 45–55 min after pairings: 164 ± 17 pA; variation of 11%). Bot [file Image_3.PDF]
