# Supplementary material for: Endocannabinoid-LTP Mediated by CB1 and TRPV1 Receptors Encodes for Limited Occurrences of Coincident Activity in Neocortex
Source: Front Cell Neurosci. 2018 Jul 5;12:182. doi: 10.3389/fncel.2018.00182 (PMC6041431; doi:10.3389/fncel.2018.00182)
Supplement: TABLE S1 — Absolute values of post-pre and pre-post ΔtSTDP. [file Table_1.docx]

**Supplementary Table 1: Absolute values of post-pre and pre-post Δt_STDP_**

| Pairings protocols | \|Δt_STDP_\| (ms) |
| --- | --- |
| 100 post-pre pairings  100 pre-post pairings  50 post-pre pairings  50 pre-post pairings  10 post-pre pairings  10 pre-post pairings  15 pre-post pairings (CB_1_R^+/+^ mice)  15 pre-post pairings (CB_1_R^-/-^ mice)  10 pre-post pairings + MPEP  10 pre-post pairings + nimodipine  10 pre-post pairings + i-THL  10 pre-post pairings + AM251  10 pre-post pairings + capsazepine  10 pre-post pairings + AMG9810  10 pre-post pairings + picrotoxin | 17.4±1.6, n=11  17.2±2.0, n=7  15.3±0.7, n=8  13.9±1.7, n=8  16.1±1.7, n=7  12.7±1.4, n=15  9.8±0.8, n=5  10.4±0.9, n=6  13.4±1.4, n=10  11.1±1.2, n=7  13.91.1, n=8  13.0±1.1, n=8  16.8±2.0, n=8  15.7±0.7, n=7  13.9±1.2, n=7 |

|Δt_STDP_| values did not display significant variations among experimental groups for post-pre or pre-post pairing protocol (one-way ANOVA: F=1.782; p=0.0968, Dunnett’s multiple comparisons test).
